# Enlarged subarachnoid space on cranial ultrasound in preterm infants: Neurodevelopmental implication

**DOI:** 10.1038/s41598-019-55604-x

**Published:** 2019-12-13

**Authors:** Sook Kyung Yum, Soo Ah Im, Yu Mi Seo, In Kyung Sung

**Affiliations:** 1 Department of Pediatrics, College of Medicine, The Catholic University of Korea, Seoul, Republic of Korea; 2 Department of Radiology, College of Medicine, The Catholic University of Korea, Seoul, Republic of Korea

**Keywords:** Paediatric research, Risk factors

## Abstract

The role of enlarged subarachnoid space (ESS) in preterm infants has not been described in concrete. We aimed to evaluate whether ESS should be considered a risk factor potentially associated with adverse neurodevelopmental outcomes in prematurity. Electronic medical records of 197 preterm infants (median 32.1 weeks' gestation) including cranial ultrasound (cUS) images, head circumferences, and Korean Developmental Screening Tests for Infants and Children (K-DST) results at 18–24 months corrected age were reviewed. The clinical characteristics and K-DST results were compared in infants with and without ESS (sinocortical width > 3.5 mm). A multivariable logistic regression analysis was performed to identify potential risk factors associated with positive K-DST results. At a median corrected age of 39.0 weeks, 81/197 (41.1%) infants presented ESS. A significantly greater percent of infants in the ESS group screened positive on the K-DST than in the no ESS group (27.2% vs 12.1%, *p* = 0.007). Within the ESS group, micro-/macrocephaly at term-equivalent age was not different with regard to the K-DST results. From the multivariable logistic regression analysis, gestational age (*p* = 0.016, OR = 0.855, 95% CI = 0.753–0.971) and ESS (*p* = 0.019, OR = 1.310, 95% CI = 1.046–1.641) were two significant risk factors associated with positive K-DST results. ESS identified on cUS at term-equivalent age in preterm infants is associated with possible developmental delays. Macrocephaly at term-equivalent age does not guarantee a benign prognosis. Future studies are required to verify ESS as a potential marker for neurodevelopmental delay in preterm infants.

## Introduction

Ultrasonography (US) is a frequently used imaging tool in the neonatal intensive care unit (NICU) because it is noninvasive and portable. Cranial US (cUS) is readily used for assessing preterm infants’ brain lesions such as intraventricular haemorrhage (IVH) or periventricular leukomalacia (PVL)^1^. However, cUS has a relatively lower accuracy than brain magnetic resonance imaging (MRI)^2–5^ in defining diffuse white matter lesions or PVL without cyst or cavitation formation, which is a more common type of non-haemorrhagic intracranial disease compared to cystic PVL^6^.

White matter injuries on MRI at term-equivalent age in very preterm infants are reportedly associated with grey matter abnormalities (including reduction in cerebral cortical grey matter volume) and future neurological impairment^7,8^. However, periventricular echogenicity greater than that of the choroid plexus, or inhomogeneous flares, which is often considered a possible US finding of non-cystic PVL^9,10^, has presented inconsistent association with punctate or diffuse white matter injuries or decreased brain volumes on MRI at term-equivalent age^11–13^ or future adverse neurological outcomes^14–16^. Rather, other combined cUS findings supportive of cerebral atrophy showed an association with neurodevelopmental outcome in previous literature^14^, with enlarged subarachnoid space (ESS) being one of them.

Structural changes around the subarachnoid space^17,18^ can be assessed easily using cUS images. An ESS may often be encountered in otherwise normally developing infants^19,20^ but is at times deemed pathologic for its potential association with adverse neurodevelopmental outcomes. Whether ESS is merely a normal variant (benign ESS) or not (e.g., brain atrophy) may be distinguished with supportive information such as the head circumference. For instance, infants with benign ESS often have macrocephaly, but those with cerebral atrophy would have blunted growth of head circumference^21^. However, the trend of head growth may not be evident in the very early days, when ESS is first identified. Furthermore, the independent role of ESS in prematurity has not been studied extensively. Therefore, we aimed to evaluate whether ESS should be considered a risk factor potentially associated with adverse neurodevelopmental outcomes in prematurity.

## Materials and Methods

### Patient selection and data collection

Electronic medical records including cUS images of all preterm infants who had been admitted to the NICU of our institute from March 2014 to June 2017 were retrieved. Infants who died during NICU stay or after hospital discharge, who did not visit for neurodevelopmental assessment at 18–24 months corrected age at our hospital, or who had major congenital anomaly or chromosomal anomaly were excluded. Infants who did not undergo repeat cUS beyond the 4th week of life were excluded, because we judged that images obtained from such infants could not be qualified for evaluation of subarachnoid space changes over time. Infants with severe IVH (grade 3 or 4), cystic PVL, and other parenchymal lesions were excluded to avoid the possible confounding effects of such lesions when evaluating the association with neurodevelopmental outcomes. This study was approved by the institutional review board of Seoul St. Mary’s Hospital. The research was performed in accordance with the Declaration of Helsinki. The need for informed consent was waived due to the retrospective nature of the study.

Three hundred and twenty-seven preterm infants who survived and visited for neurodevelopmental assessment at 18–24 months corrected age were included. Infants (n = 129) with the following were excluded: insufficient brain image data (n = 65), severe IVH (n = 36), cystic PVL (n = 14), focal parenchymal lesions (n = 12), agenesis of septum pellucidum (n = 1), and neurofibromatosis type 1 (n = 1). One infant was further excluded because the neurodevelopmental assessment was incomplete. Thus, 197 infants (108 male, median 32.1 weeks’ gestation) were finally included in the study.

Basic clinical characteristics such as gestational age (GA), birthweight, 1- and 5-minute Apgar scores, sex, mode of delivery, preterm premature rupture of membrane >18 h, mother’s age and underlying maternal morbidities (diabetes, hypertensive disorder), neonatal morbidities [respiratory distress syndrome, patent ductus arteriosus requiring treatment, necrotizing enterocolitis ≥stage 2 based on the modified Bell’s criteria^22^, moderate to severe bronchopulmonary dysplasia (BPD) based on a previous definition^23^, culture-proven sepsis], and length of NICU stay were recorded. Head circumferences at birth, term-equivalent age, and 18–24 months corrected age were collected. The measurements were categorised into percentile groups to assess the presence of microcephaly (<10^th^ percentile) and macrocephaly (>90^th^ percentile) based on the growth curves derived from Korean infants (head circumferences at birth and at term-equivalent age)^24^ and the 2017 Pediatrics and Adolescents Growth Standards provided by the Korea Centre for Disease Control and Prevention (KCDC) (head circumferences at 18–24 months corrected age)^25^.

### cUS image and measurement obtainment

The cUS images were acquired and reviewed by one paediatric radiologist with 17 years of experience. During the study period, US examinations were performed using a 4- to 10-MHz linear probe and an Acuson Sequoia 512 (Siemens Medical Engineering group, Mountain View, CA) or a 5- to 8-MHz curved probe and an Affiniti 50 G scanner (Philips Ultrasound, Bothell, Seattle, WA). The anterior fontanelle was used as an acoustic window, and images were recorded in at least six coronal and five sagittal planes. cUS examinations with additional mastoid fontanelle views were performed between the 1st and 14th day of life. The routine practice for cUS assessments in our NICU was to obtain the first cUS image within the first week of life, and follow-up schedules were arranged depending on the previous cUS results or the clinical condition of the infant, at the on-duty neonatologists’ discretion. In general, the preterm infants were followed up at a 1–2 week(s)' intervals until 4 weeks of life, and then followed up at 2 or more weeks’ intervals if IVH was less than grade 3. Follow-up cUS was withheld at approximately term-equivalent age or 1-month corrected age provided that there was no IVH or grade 1–2 IVH was involuting. Whenever a massive blood loss was suspected or when an infant underwent critical procedures such as surgery under general anaesthesia, an additional follow-up cUS was performed.

**Figure 1:**
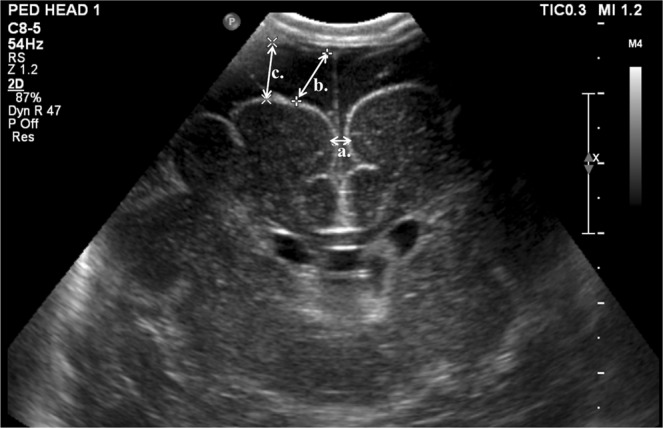
An example of cranial ultrasound image of the subarachnoid space captured via the coronal view ((**a**) IHW, interhemispheric width; (**b**) SCW, sinocortical width; (**c**) CCW, craniocortical width).

**Figure 2:**
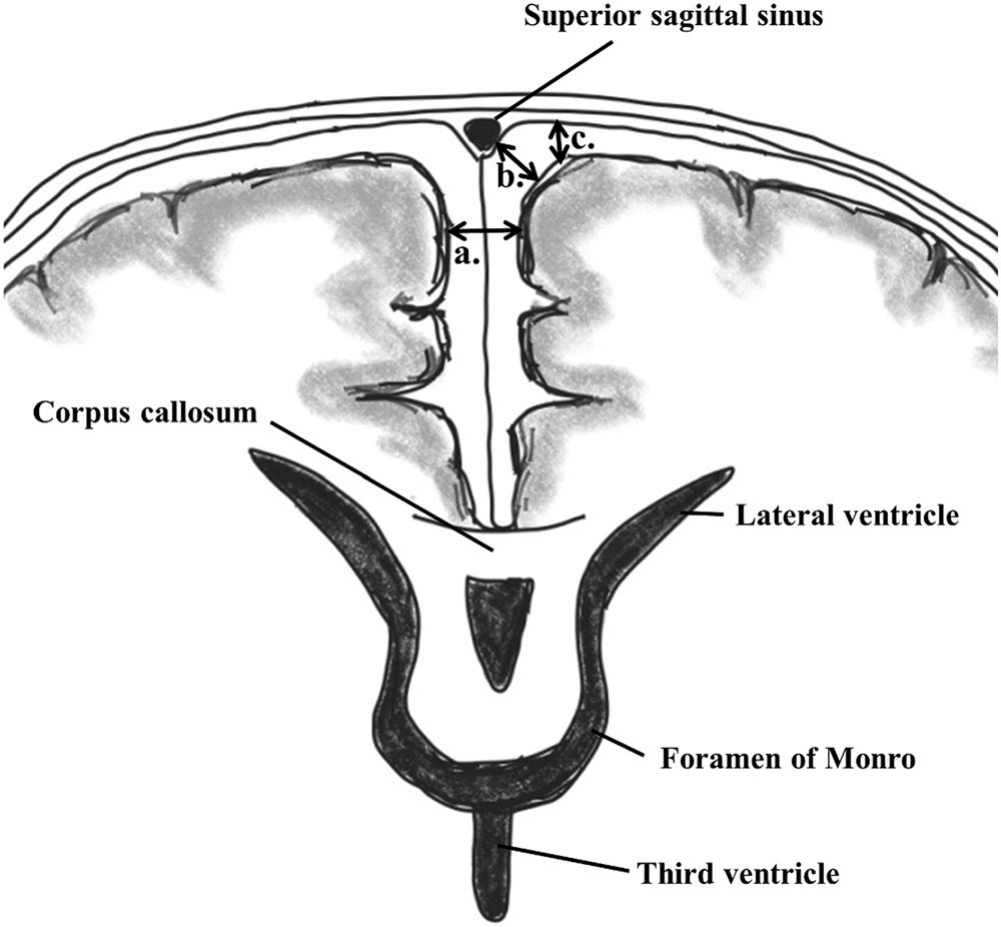
Schematic drawing of the subarachnoid space captured via the coronal view using cranial ultrasound ((**a**) IHW, interhemispheric width; (**b**) SCW, sinocortical width; (**c**) CCW, craniocortical width).

**Figure 3:**
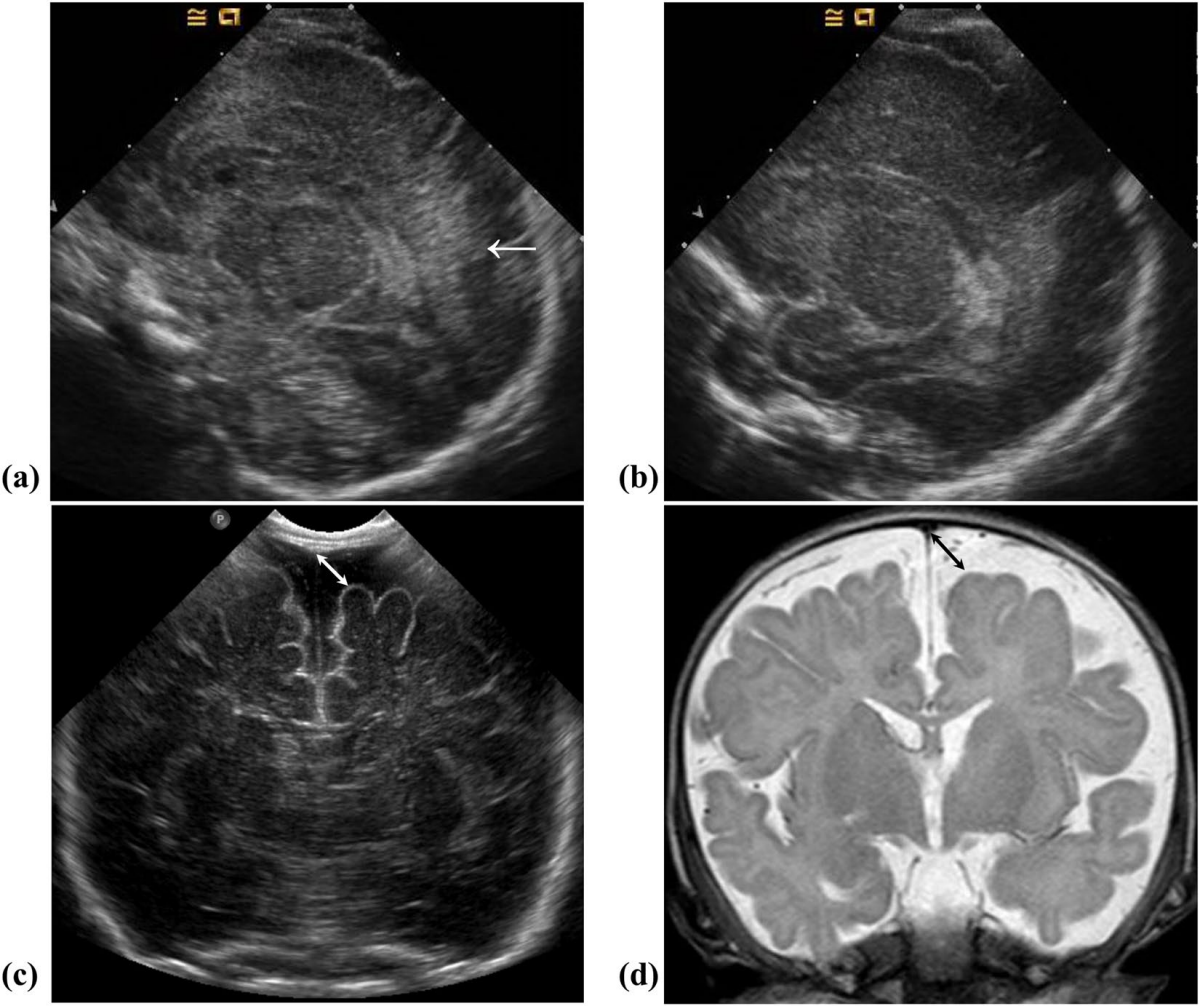
(**a**–**d**) Serial brain images of a preterm infant enrolled in our study. The infant was born at a gestational age of 29 + 5 weeks, with a birthweight of 1306 g. The initial cranial ultrasound done on the 4^th^ day of life reveals inhomogeneously increased periventricular echogenicity (arrow) to the same degree as choroid plexus (**a**). In the subsequent cranial ultrasound done at the 20^th^ day of life, the periventricular increased echogenicity is regressed without cystic formation (**b**). Enlarged subarachnoid space is first noticed on the follow-up cranial ultrasound done at 45^th^ day of life (arrow), at a corrected age of 36 + 0 weeks (sinocortical width = 8.3 mm) (**c**). On the brain magnetic resonance image (coronal T2-weighted image, TR/TE 5482/100) done at term-equivalent age, the enlarged subarachnoid space (arrow) is also noticed (**d**). The infant was screened positive in the gross motor function field at the neurodevelopmental assessment done at the follow-up visit.

IVH was graded according to Papile’s criteria^26^, and periventricular echogenicity was considered to be present when it was greater than the choroid plexus echogenicity or was inhomogeneous^9,10,27^. The subarachnoid space was measured on the coronal plane at the level of the foramen of Monro (Fig. 1) and ESS was defined based on the work by Armstrong *et al*. as sinocortical width (SCW) >3.5 mm^18^ at any age at which cUS took place. Along with SCW, interhemispheric width (IHW) and craniocortical width (CCW) – the criteria originally described previously^28^ – were also measured (Fig. 2). SCW was selected as the parameter to define ESS in our study because we deemed that CCW measurements, more easily influenced by the pressure of the probe delivered by the examiner on the anterior fontanelle, would provide inconsistent measurements. One paediatric radiologist at our institute reviewed the archived images to obtain measurements at two separate time points at least 2 weeks apart, and the mean value was selected for analyses in order to minimise intraobserver variability. An example of the series of cUS images and a brain MRI at term-equivalent age reviewed in one patient is presented on Fig. 3(a–d).

### Neurodevelopmental assessment

The neurodevelopmental assessment of the infants at 18–24 months corrected age was performed using the Korean Developmental Screening Test for Infants and Children (K-DST), developed in September 2014 by the Korean Pediatric Society and presided by the KCDC^29^. The K-DST was developed to take into account the components of the culture and infant care environment in Korea and improve detection of suspected developmental delay^30^. The K-DST is composed of questionnaires for six domains (gross motor function, fine motor function, cognition, language, social interaction, and self-help). The summed score for each domain is categorised into one of the following four: higher-than-peer level (≥1 SD), peer level (<1 SD and ≥−1 SD), follow-up evaluation is recommended (<−1 SD and ≥−2 SD), and detailed evaluation is warranted (<−2 SD). If the score for any one of the six domains is <−2 SD, the infant is considered ‘screen positive’ for the K-DST, signifying that the infant must be further evaluated for possible developmental delay. Two and five supplemental key questions associated with abnormal features (e.g., No independent walking – yes or no?) are provided at the end of the K-DST for 18 and 24 months corrected age, respectively. If the response is positive (=‘abnormal’) in any one of the key questions, the infant is considered ‘screen positive,’ regardless of the scores in the six domains. Additionally, the gross motor function status was assessed using the Gross Motor Function Classification System (GMFCS)^31^. If an infant had visited for assessment more than once during the period of 18–24 months corrected age, the most recent assessment result was included.

### Statistical analysis

The enrolled infants were divided into two groups according to the presentation of ESS. Baseline characteristics, other brain lesions identified from cUS, and the K-DST results at 18–24 months corrected age were compared between the two groups.

The infants were also divided into two groups according to the K-DST result [‘screen positive’ (score < −2 SD in any domain) vs ‘screen negative’ (score > −2 SD in all domains)]. The two groups were compared for baseline characteristics, differences in the frequency of ESS, periventricular echogenicity, or IVH, and the specific parameters of ESS were also compared.

Continuous variables were analysed using the Mann-Whitney U test, and categorical variables were analysed using the chi-square test or Fisher’s exact test, as appropriate. A multivariable logistic regression analysis was carried out in a backward conditional manner to evaluate the usefulness of parameters associated with positive results from the K-DST. *p* < 0.050 was considered statistically significant.

## Results

Eighty-one infants (41.1%) presented with ESS while 36 infants (18.3%) were ‘screen- positive’ on the K-DST.

Basic clinical characteristics were compared between the infants with and without ESS (Table 1). The ESS-group infants were born at a shorter GA (30.1 [27.9–33.4] vs 32.9 [30.5–34.4] weeks, *p* < 0.001) and a lower birthweight (1360 [1029–2033] vs 1800 [1256–2195] g, *p* = 0.003), with a significantly lower 1-minute Apgar score. The proportion of infants born via caesarean delivery was significantly greater in the ESS group (74 [91.4%] vs 93 [80.2%], *p* = 0.032). Maternal characteristics did not differ. The prevalence of moderate to severe BPD (35.8 vs 19.8%, *p* = 0.012) and the length of hospital stay (42 [21–61] vs 24 [12–48] days, *p* < 0.001) were higher and longer in the ESS group.

**Table 1:** Basic Demographics of the Included Infants Depending on the Presentation of ESS.

	No ESS (N = 116)	ESS (N = 81)	p-value
Gestational age (weeks)	32.9 [30.5–34.4]	30.1 [27.9–33.4]	<0.001
Birthweight (g)	1800 [1256–2195]	1360 [1029–2033]	0.003
1-minute Apgar score	6 [4–6]	4 [2–6]	0.008
5-minute Apgar score	7 [6–8]	7 [6–8]	0.272
Male sex	68 (58.6)	40 (49.4)	0.200
Caesarean delivery	93 (80.2)	74 (91.4)	0.032
pPROM >18 h	14 (12.1)	15 (18.5)	0.209
Mother’s age (years)	33 [31–35]	33 [32–35]	0.595
Maternal diabetes	9 (7.8)	5 (6.2)	0.670
Maternal hypertensive disorder	14 (12.1)	13 (16.0)	0.424
Respiratory distress syndrome	76 (65.5)	60 (74.1)	0.201
Surfactant instillation ≥2 times	12 (10.3)	14 (17.3)	0.157
PDA requiring treatment	9 (7.8)	13 (16.0)	0.069
Moderate to severe BPD	23 (19.8)	29 (35.8)	0.012
NEC stage ≥2	2 (1.7)	4 (4.9)	0.231*
Culture-proven sepsis	9 (7.8)	4 (4.9)	0.433
Length of stay (d)	24 [12–48]	42 [21–61]	<0.001

*Fisher’s exact test

Abbreviations: BPD, bronchopulmonary dysplasia; ESS, enlarged subarachnoid space; NEC, necrotising enterocolitis; PDA, patent ductus arteriosus; pPROM, preterm premature rupture of membrane.

Values are presented as number (percentage) or median [interquartile range].

The cUS findings compared depending on presence of ESS are described in Table 2. ESS presented at a median [interquartile range] corrected age of 39.0 [36.6–41.4] weeks. Overall, the IVH grade did not differ between the two groups, but periventricular echogenicity was more frequently found in the ESS group (12.3% vs 2.6%, *p* = 0.007). The IHW, CCW, and SCW were all significantly greater in the ESS group.

**Table 2:** Cranial Ultrasonographic Findings Compared Between Groups Divided According to the Presentation of ESS.

	No ESS (N = 116)	ESS (N = 81)	p-value
**Intraventricular haemorrhage**			0.100
None	33 (28.4)	19 (23.5)	
Grade 1	39 (33.6)	19 (23.5)	
Grade 2	44 (37.9)	43 (53.1)	
**Periventricular echogenicity**	3 (2.6)	10 (12.3)	0.007
Echogenicity greater than choroid plexus	1 (0.9)	6 (7.4)	0.020*
**Initial ESS presentation**			
Postnatal age (d)	—	57 [38–77]	—
Corrected age (weeks)	—	39.0 [36.6–41.4]	—
**ESS parameters from traditional criteria**			
Interhemispheric width (mm)	2.6 [1.8–3.3]	4.8 [3.5–5.9]	<0.001
Craniocortical width (mm)	1.5 [1.0–2.1]	4.2 [3.3–4.8]	<0.001
Sinocortical width (mm)	2.0 [1.6–2.6]	4.6 [4.1–5.5]	<0.001

*Fisher’s exact test.

Abbreviations: ESS, enlarged subarachnoid space.

Values are presented as number (percentage) or median [interquartile range].

The K-DST results assessed at 18–24 months corrected age were compared between the infants depending on the presence of ESS (Table 3). The proportion of ‘screen-positive’ infants was more than twice higher in the ESS group than in the no ESS group (27.2% vs 12.1%, *p* = 0.007). The difference was most evident in the gross motor function domain (23.5% vs 6.0%, *p* < 0.001). Concerning the GMFCS, a greater percentage of infants in the ESS group showed level ≥2 (7.4 vs 0.9%, *p* = 0.020), of which one infant in the ESS group was at level 3. All infants with positive responses on the supplementary questions or GMFCS had a score <−2 SD in at least one of the six domains of the K-DST.

**Table 3:** Neurodevelopmental Screening Test Results Assessed at 18 to 24 Months Corrected Age Compared Between Groups Divided According to the Presence of ESS.

	No ESS (N = 116)	ESS (N = 81)	p-value
<−**2SD in any domain**	14 (12.1)	22 (27.2)	0.007
<−2SD in gross motor domain	7 (6.0)	19 (23.5)	<0.001
<−2SD in fine motor domain	4 (3.4)	7 (8.6)	0.205*
<−2SD in cognition domain	2 (1.7)	9 (11.1)	0.008*
<−2SD in language domain	4 (3.4)	10 (12.3)	0.017
<−2SD in sociality domain	3 (2.6)	6 (7.4)	0.165*
<−2SD in self-help domain	8 (6.9)	10 (12.3)	0.192
‘**Yes**’ **in any one of the key supplementary questions**	0 (0.0)	7 (8.6)	0.002*
**GMFCS level** ≥ **2**	1 (0.9)	6 (7.4)	0.020*

*Fisher’s exact test.

Abbreviations: ESS, enlarged subarachnoid space; GMFCS, Gross Motor Function Classification System; SD, standard deviation.

Values are presented as number (percentage).

The infants were compared for baseline characteristics depending on the K-DST result (Table 4). The screen-positive group showed significantly shorter GA (29.7 [26.4–32.6] vs 32.7 [29.9–34.1] weeks, *p* < 0.001), lower birthweight (1152 [860–1803] vs 1734 [1205–2152] g, *p* = 0.001), and lower 1-minute Apgar score (4 [2–6] vs 5 [3–6], *p* = 0.021). Moderate to severe BPD was the only neonatal morbidity that showed significant difference in prevalence (47.2 vs 21.7%, *p* = 0.002) between the screen-positive vs negative groups. The length of hospital stay was also significantly longer in the screen-positive group.

**Table 4:** Basic Demographics of the Included Infants Depending on the K-DST Result.

	Screen negative (n = 161)	Screen positive (n = 36)	p-value
Gestational age (weeks)	32.7 [29.9–34.1]	29.7 [26.4–32.6]	<0.001
Birthweight (g)	1734 [1205–2152]	1152 [860–1803]	0.001
1-minute Apgar score	5 [3–6]	4 [2–6]	0.021
5-minute Apgar score	7 [6–8]	7 [5–8]	0.407
Male sex	88 (54.7)	20 (55.6)	0.922
Caesarean delivery	135 (83.9)	32 (88.9)	0.447
pPROM >18 h	21 (13.0)	8 (22.2)	0.160
Mother age’s (years)	33 [31–35]	34 [31–37]	0.311
Maternal diabetes	13 (8.1)	1 (2.8)	0.473*
Maternal hypertensive disorder	24 (14.9)	3 (8.3)	0.424*
Respiratory distress syndrome	107 (66.5)	29 (80.6)	0.098
Surfactant instillation ≥2 times	20 (12.4)	6 (16.7)	0.585*
PDA requiring treatment	16 (9.9)	6 (16.7)	0.249*
Moderate to severe BPD	35 (21.7)	17 (47.2)	0.002*
NEC stage ≥2	4 (2.5)	2 (5.6)	0.302*
Culture-proven sepsis	11 (6.8)	2 (5.6)	>0.999*
Length of stay (d)	26 [13–50]	56 [28–89]	<0.001

*Fisher’s exact test.

Abbreviations: BPD, bronchopulmonary dysplasia; ESS, enlarged subarachnoid space; K-DST, Korean Developmental Screening Test for Infants and Children; NEC, necrotising enterocolitis; PDA, patent ductus arteriosus; pPROM, preterm premature rupture of membrane.

Values are presented as number (percentage) or median [interquartile range].

Table 5 describes the prevalence of ESS and periventricular echogenicity, and the IHW, CCW, and SCW compared between the ‘screen-positive’ and ‘screen-negative’ groups. Significantly more infants had ESS in the ‘screen-positive’ group (61.1% vs 36.6%, *p* = 0.007), initially presenting at term-equivalent age in both groups. IHW, CCW, and SCW were significantly greater in the ‘screen-positive’ group.

**Table 5:** Cranial Ultrasonographic Findings Compared in Infants Depending on the K-DST Result.

	Screen negative (≥−2SD) (N = 161)	Screen positive (<−2SD) (N = 36)	p-value
**Initial ESS presentation**			
Postnatal age (d)	46 [30–71]	60 [44–116]	0.026
Corrected age (weeks)	38.4 [35.1–41.1]	37.9 [35.3–41.4]	0.789
**Presence of ESS**	59 (36.6)	22 (61.1)	0.007
**Interhemispheric width** (**mm**)	3.1 [2.1–4.6]	4.4 [2.3–5.9]	0.044
**Craniocortical width** (**mm**)	2.2 [1.4–3.5]	3.4 [2.0–4.7]	0.007
**Sinocortical width** (**mm**)	2.7 [1.7–4.2]	4.2 [2.4–5.7]	0.003
**Periventricular echogenicity**	10 (6.2)	3 (8.3)	0.709*
More hyperechoic than choroid plexus	5 (3.1)	2 (5.6)	0.614*

*Fisher’s exact test.

Abbreviations: ESS, enlarged subarachnoid space; K-DST, Korean Developmental Screening Test for Infants and Children; SD, standard deviation.

Values are presented as number (percentage) or median [interquartile range].

Head circumferences measured at birth, term-equivalent age, and 18–24 months corrected age were compared between infants with and without ESS, further subdivided according to the K-DST results (Table 6). A significantly greater proportion of infants had microcephaly in the K-DST-positive subgroup (27.3 vs 7.3%, *p* = 0.028) in the ESS group but not in the no-ESS group. The prevalence of macrocephaly at neither term-equivalent age nor 18–24 months corrected age showed statistically significant difference between the subgroups within the ESS and no ESS groups.

**Table 6:** Presence of Micro- or Macrocephaly at Birth and Term-Equivalent Age Depending on the Result of the K-DST in the ESS and no ESS Group Infants.

Head circumference	No ESS		p	ESS		p
	Screen negative (N = 102)	Screen positive (N = 14)		Screen negative (N = 59)	Screen positive (N = 22)	
**At birth**						
<10^th^ percentile	16 (15.7)	5 (35.7)	0.130*	12 (20.3)	4 (18.2)	>0.999*
≥90^th^ percentile	4 (3.9)	0 (0.0)	>0.999*	2 (3.4)	4 (18.2)	0.044*
**At term-equivalent age**						
<10^th^ percentile	16 (15.7)	3 (21.4)	0.699*	8 (13.6)	7 (31.8)	0.104*
≥90^th^ percentile	11 (10.8)	0 (0.0)	0.355*	4 (6.8)	2 (9.1)	0.661*
**At 18–24 months corrected age**^a^						
<10^th^ percentile	7 (7.2)	2 (14.3)	0.317*	4 (7.3)	6 (27.3)	0.028*
≥90^th^ percentile	14 (14.4)	1 (7.1)	0.688*	8 (14.5)	2 (9.1)	0.715*

*Fisher’s exact test.

^a^Measurements available from 188 infants (97 screen negative infants and 14 screen positive infants in the no ESS group, 55 screen negative infants and 22 screen positive infants in the ESS group).

Abbreviations: ESS, enlarged subarachnoid space; K-DST, Korean Developmental Screening Test for Infants and Children.

Values are presented as number (percentage).

A multivariable logistic regression analysis was performed to evaluate the association with positive results on the K-DST (Table 7). Significant variables from univariable analyses and clinically important factors were included. GA [*p* = 0.016, odds ratio (OR) = 0.855, 95% confidence interval (CI) = 0.753–0.971] and ESS [*p* = 0.019, OR = 1.310, 95% CI = 1.046–1.641] were two factors that maintained statistical significance.

**Table 7:** Multivariable Logistic Regression Analysis for Evaluation of the Association with Positive K-DST Results.

	p-value	Odds ratio	95% confidence interval
Intraventricular haemorrhage grade	0.080	3.280	0.868–12.397
Gestational age	0.016	0.855	0.753–0.971
1-min Apgar score	0.518	0.925	0.730–1.172
Moderate BPD	0.958	0.971	0.323–2.918
ESS	0.019	1.310	1.046–1.641
Macrocephaly at birth^a^	0.416	1.931	0.395–9.429
Microcephaly^b^ at 18–24 months CA	0.082	2.643	0.884–7.905

Abbreviations: BPD, bronchopulmonary dysplasia; CA, corrected age; ESS, enlarged subarachnoid space; K-DST, Korean Developmental Screening Test for Infants and Children.

^a^Macrocephaly: head circumference ≥90^th^ percentile.

^b^Microcephaly: head circumference <10^th^ percentile.

## Discussion

We evaluated the association between ESS and neurodevelopmental test results in preterm infants. Based on our study results, ESS was associated with positive results on the K-DST, which was most evident in the gross motor function domain. Although it failed to maintain statistical significance in the multivariable analysis, microcephaly at 18–24 months corrected age tended to be more prevalent in the K-DST screen-positive infant subgroup in the ESS group.

The ESS criteria we used in our study were based on a previous work by Armstrong *et al*.^18^ including preterm infants at <37 weeks’ GA. The traditional criteria for ESS – IHW > 6 mm, CCW > 4 mm, and SCW > 3 mm – were based on values derived from infants at 1–12 months of age^28^. Meanwhile, Lam *et al*.^32^ suggested their regression curves as the new criteria for normal upper limit values including term infants. Neither reference value has been validated in preterm infants; thus, we decided to adhere to the value proposed by Armstrong *et al*.^18^.

The ESS course has been previously described as benign^19,20,33^. However, some studies have reported the possibility of transient and, less frequently, permanent developmental delays associated with ESS^34,35^. Our study results also revealed an association with developmental delay. Whether or not these delays would be transient or permanent could not be confirmed at this stage, but it does not change the fact that these infants may potentially not be able to “grow out” of the delays and may require rehabilitative intervention of some type. Therefore, ESS in preterm infants deserves the attention of clinicians.

To our knowledge, no studies have examined how to distinguish the suspected course of ESS at the early stages of presentation in preterm infants. A concomitant presentation of ESS and macrocephaly is considered to be associated with a lower possibility of adverse neurological outcomes in full-term infants. However, based on our study results, the same cannot be determined in preterm infants. The initial presentation of ESS was identified at term-equivalent age and postnatal age of 57 [38–77] days, which is earlier than that in full-term infants who present with ESS at 3 to 8 months of life. The prevalence of macrocephaly at term-equivalent age or 18–24 months corrected age did not differ depending on the neurodevelopmental screening assessments in infants presenting with ESS. Therefore, a concomitant presentation of ESS and macrocephaly at term-equivalent age does not guarantee a normal future neurodevelopmental course in preterm infants. Rather, microcephaly at 18–24 months corrected age was significant at least in the univariable analysis. Therefore, we postulate that ESS in preterm infants is a neglected condition that deserves more attention from both clinicians and radiologists. A larger-scale prospective study is warranted to validate our results and draw specific conclusions for the role of ESS in future neurodevelopmental outcomes in preterm infants.

Our study is restricted by several factors. The retrospective design and the small number of included patients is the first limitation. Infants who resided at far distances and were lost to follow-up at our hospital were excluded. Some infants would have received follow-up evaluations at other institutes, but such information was inaccessible. Secondly, we did not analyse the results from examinations such as the Bayley Scales of Infant and Toddler Development (BSID) but used the K-DST for outcome assessment. Our unit protocol is to carry out follow-up neurodevelopmental evaluations largely based on the K-DST. If screened positive, the infant is referred for further examinations such as the BSID to the department of rehabilitative medicine, unless the infant is judged to be at a very high risk for adverse neurodevelopmental outcome (such as most extreme prematurity, severe IVH, cystic PVL, and hypoxic-ischemic encephalopathy). However, the K-DST reflects the cultural and environmental facets of our country^29,30^ and is easy to use, so complete assessment was achieved in most infants who visited the outpatient clinic at 18–24 months corrected age, which is the strength of our study. We also used the GMFCS to further complement our assessment. Another limitation is that we did not include cerebellar injury in analysing the cUS findings, because there was insufficient data in the archive. However, since large cerebellar haemorrhagic injuries often present with concomitant severe supratentorial haemorrhagic lesions^36^, those with such lesions would have been excluded per our study enrollment scheme excluding severe IVH. Furthermore, small cerebellar haemorrhages are difficult to detect with cUS^37^, so there is a high probability that patients with isolated cerebellar microhaemorrhages would not have been readily identified, even if appropriate images had been obtained. In addition, because the cUS were done at several weeks’ intervals in the later stages of life in some infants, it is possible that in some cases, the cystic stage of PVL may have been missed (as in the case of Fig. 3, in which there was a long delay between the first (a) and second (b) cUS scan). Finally, we did not analyse brain MRI findings due to lack of data. However, cUS is the most generally-used imaging modality with a fair cost-effective character. In contrast, MRI is expensive, often requires sedation, and is frequently difficult to perform in infants with critical pulmonary conditions. Therefore, using cUS findings to assess ESS is meaningful for its high applicability at clinical settings similar to our NICU.

## Conclusion

Enlarged subarachnoid space identified on cranial ultrasound at term-equivalent age is associated with possible developmental delays and could be an early ‘warning sign’ of neurodevelopmental delay in preterm infants. Concomitant presentation of enlarged subarachnoid space and macrocephaly at term-equivalent age in preterm infants may not guarantee a benign nature. Future prospective studies with a large number of subjects with sequential follow-up examinations with sufficient time intervals are needed to verify our findings.

## Data Availability

The data is available only upon a reasonable request to the corresponding author.
